# Neurotropin Inhibits Lipid Accumulation by Maintaining Mitochondrial Function in Hepatocytes via AMPK Activation

**DOI:** 10.3389/fphys.2020.00950

**Published:** 2020-08-06

**Authors:** Qinglan Wang, Zhijun Wang, Mingyi Xu, Wei Tu, I-Fang Hsin, Aleksandr Stotland, Jeong Han Kim, Ping Liu, Mitsuru Naiki, Roberta A. Gottlieb, Ekihiro Seki

**Affiliations:** ^1^Department of Medicine, Cedars-Sinai Medical Center, Los Angeles, CA, United States; ^2^E-Institute of Shanghai Municipal Education Committee, Shanghai University of Traditional Chinese Medicine, Shanghai, China; ^3^Department of Pharmacological Research, Institute of Bio-Active Science, Nippon Zoki Pharmaceutical Co., Ltd., Osaka, Japan; ^4^Department of Biomedical Sciences, Cedars-Sinai Medical Center, Los Angeles, CA, United States; ^5^Smidt Heart Institute, Cedars-Sinai Medical Center, Los Angeles, CA, United States

**Keywords:** AMPK, fatty liver, mitochondria, lipid metabolism, liver

## Abstract

The accumulation of lipid droplets in the cytoplasm of hepatocytes, known as hepatic steatosis, is a hallmark of non-alcoholic fatty liver disease (NAFLD). Inhibiting hepatic steatosis is suggested to be a therapeutic strategy for NAFLD. The present study investigated the actions of Neurotropin (NTP), a drug used for chronic pain in Japan and China, on lipid accumulation in hepatocytes as a possible treatment for NAFLD. NTP inhibited lipid accumulation induced by palmitate and linoleate, the two major hepatotoxic free fatty acids found in NAFLD livers. An RNA sequencing analysis revealed that NTP altered the expression of mitochondrial genes. NTP ameliorated palmitate-and linoleate-induced mitochondrial dysfunction by reversing mitochondrial membrane potential, respiration, and β-oxidation, suppressing mitochondrial oxidative stress, and enhancing mitochondrial turnover. Moreover, NTP increased the phosphorylation of AMPK, a critical factor in the regulation of mitochondrial function, and induced PGC-1β expression. Inhibition of AMPK activity and PGC-1β expression diminished the anti-steatotic effect of NTP in hepatocytes. JNK inhibition could also be associated with NTP-mediated inhibition of lipid accumulation, but we did not find the association between AMPK and JNK. These results suggest that NTP inhibits lipid accumulation by maintaining mitochondrial function in hepatocytes via AMPK activation, or by inhibiting JNK.

## Introduction

The prevalence of non-alcoholic fatty liver disease (NAFLD) has dramatically increased in the last decade, and NAFLD has become one of the most common health concerns associated with metabolic syndrome, affecting 25% of the adult populations in Western and Asian countries (Fan et al., 2017; Friedman et al., 2018). NAFLD is often associated with obesity, diabetes, cardiovascular disease, hypertension, and metabolic syndrome (Friedman et al., 2018) but also affects 8–19% of non-obese Asians (Fan et al., 2017). The disease spectrum of NAFLD ranges from simple steatosis to steatosis with hepatocyte ballooning, liver inflammation, and fibrosis, referred to as non-alcoholic steatohepatitis (NASH). Approximately 20–25% of NAFLD patients develop NASH, which may progress to fibrosis and cirrhosis (Friedman et al., 2018; Younossi et al., 2018). Fibrosis is the most critical determinant of the prognosis of patients with NASH. Notably, the pathological conditions of NAFLD, even without cirrhosis, significantly increase the incidence of hepatocellular carcinoma (Friedman et al., 2018). Although current phase 3 clinical trials for NAFLD/NASH are targeting specific molecular pathways, including FXR, ASK1, CCR2/CCR5, and PPARα/δ, no effective drugs have been approved by the United States Food and Drug Administration (Friedman et al., 2018). However, existing drugs, for which the safety data are available, can be repurposed for new indications, thereby saving time and cost. The goal of the present study was to investigate the potential to repurpose the drug Neurotropin (NTP) for the treatment of NAFLD through the *in vitro* model.

Neurotropin is a non-protein extract from inflamed rabbit skin following the administration of vaccinia virus. It has been widely used as an analgesic in Japan and China for more than 50 years for the treatment of chronic pain conditions, such as low back pain, cervico-omo-brachial syndrome, and frozen shoulder syndrome, and neuropathic pain conditions, such as postherpetic neuralgia and hyperesthesia of subacute myelo-optic neuropathy (Inagaki et al., 1990; Zhu et al., 2017). The analgesic effect of NTP is mediated by the activation of the descending pain inhibitory system (Okazaki et al., 2008) and by the induction of brain-derived neurotrophic factor (Ishikawa et al., 2015). Notably, NTP is also cytoprotective against neurodegeneration and neuropathy from anticancer chemotherapy-induced neurotoxicity (Kawashiri et al., 2009) and TNFα- and IL-1β-induced hepatocyte apoptosis (Zhang et al., 2014). Furthermore, NTP inhibits several inflammatory pathways, including NF-κB, JNK, ERK, and p38 MAPK pathways in neurons, microglia, and hepatocytes (Zhang et al., 2014; Nishimoto et al., 2016; Fang et al., 2017; Zheng et al., 2018), and suppresses the induction of TNFα, IL-6, and COX-2 by IL-1β and LPS in intervertebral disk cells, microglia, and hepatocytes (Yoshida et al., 2008; Zhang et al., 2014; Zheng et al., 2018). These anti-inflammatory and cytoprotective effects may be beneficial for patients with NASH, which is driven by inflammation in the liver.

One therapeutic strategy for NASH involves inhibiting the formation and accumulation of lipid droplets in the cytoplasm of hepatocytes, or hepatic steatosis (Loomba et al., 2017), which is also a hallmark of NAFLD (Zhang et al., 2014). Fatty acid β-oxidation in mitochondria is crucial for hepatic lipid metabolism (Mansouri et al., 2018). However, lipid accumulation in hepatocytes induces mitochondrial damage and stimulates the production of mitochondrial reactive oxygen species (mtROS), which exacerbates mitochondrial damage and impedes β-oxidation. A recent study demonstrated that NTP increases the mitochondrial membrane potential (MMP) and suppresses ROS production in neurons to protect them against damage induced by the amyloid β (25–35) peptide (Fang et al., 2017). Thus, we hypothesized that NTP might prevent hepatic steatosis and inflammation by preventing mitochondrial damage in hepatocytes induced by cellular stresses, such as the exposure to cytotoxic free fatty acids (FFAs). Indeed, we found that NTP inhibited lipid accumulation and mtROS production and enhanced mitochondrial respiration and β-oxidation. Intriguingly, NTP activated 5’ AMP-activated protein kinase (AMPK) and the related peroxisome proliferator-activated receptor (PPAR) γ coactivator-1β (PGC-1β), which were responsible for the beneficial effect of NTP on mitochondrial functions. The results from this study indicate that NTP may prevent NAFLD progression by inhibiting hepatocyte lipid accumulation via activating AMPK and maintaining mitochondrial quality.

## Materials and Methods

### Reagents

Neurotropin (NTP) was provided by Nippon Zoki Pharmaceutical Co., Ltd (Osaka, Japan). Tetramethylrhodamine (TMRM) was purchased from Anaspec (Fremont, CA, United States). MitoSOX red was purchased from Invitrogen (Carlsbad, CA, United States). Sodium palmitate, linoleic acid sodium salt, the AMPK inhibitor (Compound C; 6-[4-(2-Piperidin-1-ylethoxy) phenyl]-3-pyridin-4-ylpyrazolo [1,5-a]pyrimidine, Sigma-Aldrich, St. Louis, MO, United States), SP600126 (JNK inhibitor), and the antibody for β-actin were purchased from Sigma-Aldrich (St. Louis, MO, United States). TG kits were from Pointe Scientific Inc (Canton, MI, United States). Antibodies against AMPK, phospho-AMPK, JNK, and phospho-JNK were purchased from Cell Signaling (Danvers, MA, United States). Control scramble siRNA (#12935300) and *Ppargc1b* siRNA (#AM16708) were purchased from Thermo Fisher Scientific (Waltham, MA, United States).

### Hepatocyte Isolation and Treatment

Primary hepatocytes were isolated from wild-type C57BL/6 mice (Jackson Laboratory, Bar Harbor, ME, United States) using the *in situ* collagenase perfusion method described previously (Yang et al., 2013) and seeded on collagen I (BD Biosciences, San Jose, CA, United States) coated plates. All mice received humane care according to the National Institutes of Health recommendations outlined in their Guide for the Care and Use of Laboratory Animals. All animal experiments were approved by Cedars-Sinai Medical Center Institutional Animal Care and Use Committee. Cells with >90% viability were used for the experiments. Cells were cultured in Dulbecco’s modified Eagle medium (DMEM) containing 10% fetal bovine serum (FBS) for 4 h. The concentrations of NTP used in this study have been determined in our previous study that showed the anti-apoptotic effect of NTP (Zhang et al., 2014). For experiments using siRNAs, scramble siRNA (50 nM) or *Ppargc1b* siRNA (50 nM) was transfected using lipofectamine RNAiMAX (Thermo Fisher Scientific, Waltham, MA, United States) according to the manufacturer’s protocol. To confirm the efficacy of the knockdown of *Ppargc1b*, we measured mRNA levels after 24 h of the transfection by qPCR, which was shown in Figure 7A. After 24 h of the transfection, the cells were treated with NTP (0.2 or 0.4 NTP units:NU/ml) or compound C (5 μM) (0.05% DMSO was used as vehicle control) (Liu et al., 2014) for 1 h followed by 200 μM PA or 12 μM LA for an additional 24 h. The bioactive concentration of compound C was determined in the previous study (Liu et al., 2014). For other experiments, 4 h later, the medium was changed to serum-free DMEM overnight, and the cells were then treated with NTP (0.2 or 0.4 NU/ml), compound C (5 μM), or SP600125 (20 μM) for 1 h, followed by 200 μM PA or 12 μM LA for an additional 24 h.

### Oil Red O Staining

After treatment, primary hepatocytes were washed twice with phosphate-buffered saline (PBS), fixed with 10% neutral-buffered formalin for 1 h, and then stained with Oil red O solution for 10 min at room temperature, followed by washing in H_2_O three times. Cell images were captured with a Leica DMi8. After image capturing, isopropanol was added to each well, and the extracted dye was measured at 500 nm for quantification. The Oil red O values were normalized to the optical density (OD) of MTT [3-(4,5-dimethyl-2-thiazolyl)-2,5-diphenyl-2H-tetrazolium bromide].

### Total Cellular TG Content Measurement

Total cellular TG levels of primary hepatocytes were determined using TG GPO liquid reagent sets (Pointe Scientific Inc.) according to the manufacturer’s protocol. The total protein concentration was measured by BCA (Thermo Fisher Scientific, Waltham, MA, United States) assay according to the manufacturer’s protocol. TG content was normalized to the total protein concentration and expressed as micrograms per micrograms protein.

### RNA-Seq Sample Preparation and Sequencing

Total RNA was extracted from primary hepatocytes using TRIzol and purified using a NucleoSpin RNA kit according to the manufacturer’s instructions. RNA concentration, purity, and integrity were measured with an Agilent 2100 BioAnalyzer (Agilent Technologies). Beijing Genomics Institute performed the RNA sequencing, as previously described (Lv et al., 2016). RNA libraries were prepared for sequencing via an Illumina HiSeq 4000 using standard BGISEQ-500 protocols.

### RNA-Seq Data Analysis

HISAT (Kim et al., 2015) and Bowtie2 (Langmead et al., 2009) were applied to align sequencing reads to the reference gene and reference genome, respectively. The relative gene expression [reads per kilobase per million (RPKM)] was quantified using RSEM (Li and Dewey, 2011). The NOISeq non-parametrical statistical method (Tarazona et al., 2011, 2015; Carcel-Trullols et al., 2012; Chen et al., 2013; Durban et al., 2013; Ferreira et al., 2013; Liu et al., 2013; Shearman et al., 2013; Xia et al., 2013; Zhu et al., 2013; Su et al., 2014) was used to screen the differentially expressed genes (DEGs) between two groups according to the following default criteria: fold change of ≥1.5 and diverge probability of ≥0.95. A Venn diagram was used to depict common DEGs between different groups. The commonly changed genes by NTP in PA- and LA-treated cells were subjected to GO analysis using the David database (Huang Da et al., 2009). A DEG-GO term network was constructed using Cytoscape v3.1.1. The heatmap of the expression profile of DEGs was constructed by multiExperiment Viewer (Mev) v4.6 software. RNA-seq data have been uploaded to the GEO (accession no. GSE132251).

### Measurement of MMP

Mitochondrial membrane potential was examined by using TMRM, a cell-permeant, cationic red fluorescent dye. After treatment, primary hepatocytes were incubated in DMEM containing TMRM (100 nM) for 30 min, followed by washing with PBS twice. TMRM fluorescence intensity was measured by a microplate reader at excitation/emission wavelengths of 488/560 nm. The values were normalized to the OD of MTT.

### Measurement of mtROS Production

Mitochondrial reactive oxygen species production was measured by MitoSOX red (Invitrogen) according to the manufacturer’s protocol. Briefly, after treatment, primary hepatocytes were washed once with Hanks balanced salt solution and then incubated with MitoSOX red for 15 min, followed by washing twice with PBS. The fluorescence intensity was measured by a microplate reader at excitation/emission wavelengths of 510/580 nm. The values were normalized to the OD of MTT.

### Measurement of Mitochondrial Respiration

Mitochondrial respiration was examined by measuring the OCR using the Seahorse XF24 extracellular flux analyzer (Seahorse Bioscience, Billerica, MA, United States). Primary hepatocytes were seeded in DMEM containing 10% FBS at a density of 2 × 10^4^ cells/well in XF24 cell culture plates (Seahorse Bioscience). Four hours later, the cells were treated with NTP in serum-free medium for 24 h at 37°C with 5% CO_2_. Before the assay was started, the medium was replaced with bicarbonate-free low-buffered medium (Seahorse Bioscience) containing 25 mM glucose, 2 mM glutamine, 1 mM sodium pyruvate (pH 7.4) and the plates were preincubated in the absence of CO_2_ at 37°C. After calibration, the plates were placed into the XF24 extracellular flux analyzer, and the OCR was evaluated by sequential injection of 1 μM oligomycin, 1 μM carbonyl cyanide-4-(trifluoromethoxy) phenylhydrazone (FCCP), and 0.5 μM rotenone/antimycin A. Then OCR was automatically calculated by the Seahorse XF24 analyzer. The OCR values were normalized to total protein concentration.

### Measurement of FAO

Fatty acid oxidation was measured using the XF PA-bovine serum albumin (BSA) FAO substrate with the XF Cell Mito stress test according to the manufacturer’s protocol. Primary hepatocytes were seeded in DMEM containing 10% FBS at a density of 2 × 10^4^ cells/well in XF24 cell culture plates (Seahorse Bioscience). Four hours later, the culture medium was replaced with a substrate-limited medium with or without NTP. Twenty-four hours later, the medium was replaced with FAO assay medium and incubated in the absence of CO_2_ at 37°C for 45 min. Then, the plate was placed into the XF24 extracellular flux analyzer, and the OCR was evaluated by sequential injection of palmitate-BSA or BSA (175 μM), oligomycin (5 μM), FCCP (8 μM), and rotenone/antimycin A (0.5 μM). Then OCR was automatically calculated by the Seahorse XF24 analyzer. The OCR values were normalized to total protein concentration.

### Examination of Mitochondrial Turnover by MitoTimer

Mitochondrial turnover was analyzed by measuring the green and red fluorescence intensities in HepG2 cells stably transfected with pMitoTimer (Hernandez et al., 2013). HepG2 cells were pretreated with NTP for 1 h, followed by treatment with 200 μM PA or 12 μM LA for another 24 h. For siRNA experiments, the cells were incubated with scramble siRNA or 50 nM *Ppargc1b* siRNA for 24 h before NTP treatment. The green fluorescence intensity (476 nm) and red fluorescence intensity (589 nm) were measured by a fluorescent microplate reader. The values were normalized to the OD of MTT.

### Western Blot Analysis

The cells were harvested and homogenized in a lysis buffer [50 mM Tris–HCl (pH 8.0), 5 mM EDTA, 150 mM NaCl, 0.5% Nonidet P-40, 0.5 mM phenylmethylsulfonyl fluoride, and 0.5 mM dithiothreitol] for 30 min at 4°C. The protein concentration was determined using a bicinchoninic acid protein assay kit (Thermo Scientific). Equal amounts of protein (30 μg) were separated by 10% sodium dodecyl sulfate-polyacrylamide gel electrophoresis and then transferred to a nitrocellulose membrane. The membrane was blocked with 5% BSA/PBS at room temperature for 1 h and then incubated with primary antibody at 4°C overnight, followed by incubation with a secondary antibody conjugated to horseradish peroxidase. The immunocomplexes were visualized with enhanced chemiluminescence kits (Amersham Biosciences). The band intensity was quantified by Image J software (NIH). Four biologically independent samples/group were used for the quantification.

### qPCR

Total RNA from cells was extracted using TRIzol RNA isolation reagent (Invitrogen) and reverse-transcribed to cDNA using a high-capacity cDNA reverse transcription kit (Applied Biosystems). qPCR was performed using iTaq Univer SYBR green Supermix 1000 (Bio-Rad) on a CFX96 real-time PCR system (Bio-Rad). The mouse-specific primers for qPCR are as follows: *Cox7c* forward primer, 5′-ATG TTG GGC CAG AGT ATC CG-3′; *Cox7c* reverse primer, 5′-ACC CAG ATC CAA AGT ACA CGG-3′; *Cox8a* forward primer, 5′-TTC CTG CTT CGT GTG TTG TC-3’; *Cox8a* reverse primer, 5′-GAT TGC AGA AGA GGT GAC TGG-3′; *Ppargc1b* forward primer, 5′-TCC TGT AAA AGC CCG GAG TAT-3′; *Ppargc1b* reverse primer, 5′-GCT CTG GTA GGG GCA GTG A-3′; β-actin forward primer, 5′-GGC TGT ATT CCC CTC CAT CG-3′; β-actin reverse primer, 5′- CCA GTT GGT AAC AAT GCC ATG T-3′. β-actin was used as an internal control for normalization.

### Statistical Analysis

Statistical analyses were performed using GraphPad Prism 7.00 software. Data are expressed as means ± standard errors of the means. Differences between the two groups were compared using a two-tailed unpaired Student’s *t*-test. Differences between multiple groups were compared using one-way analysis of variance, followed by Tukey’s *post hoc* analysis. *P-*values of <0.05 were considered significant.

## Results

### NTP Inhibits FFA-Induced Lipid Accumulation in Hepatocytes

**FIGURE 1:**
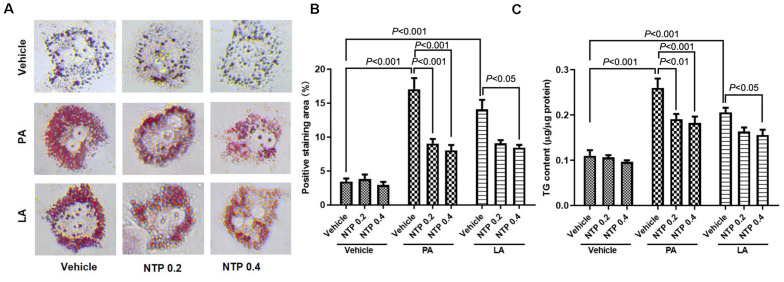
Effects of NTP on PA- and LA-induced lipid accumulation in hepatocytes. Primary hepatocytes were pretreated with NTP (0.2 or 0.4 NU/mL) for 1 h, followed by treatment with 200 μM palmitate (PA) or 12 μM linoleate (LA) for an additional 24 h. Cellular lipid accumulation was examined by Oil red O staining and measurement of cellular triglyceride (TG) content. **(A)** Representative microscopic images of Oil red O staining. **(B)** Quantification of Oil red O staining. Data are presented as the means ± standard errors of the means of 10 high power fields (×200); **(C)** cellular TG content. The values were normalized to total protein concentrations. Data are represented as mean ± SEM of triplicate. Similar results were obtained in three independent experiments. Representative results are shown.

We first investigated the protective effect of NTP against FFA-induced lipid accumulation in primary hepatocytes. Prior to the experiments, we validated whether NTP has a cytotoxic effect on primary mouse hepatocytes. We treated primary hepatocytes with NTP at the concentrations ranging from 0.1 to 1.6 NU/mL and did not find the hepatocytotoxic effect of NTP even at 1.6 NU/mL (Supplementary Figure S1). We decided to use the concentrations of NTP at 0.2 and 0.4 NTP NU/mL based on the concentrations that show the anti-apoptotic effect in our previous study (Zhang et al., 2014). Palmitate (PA) and linoleate (LA) are two major FFAs that accumulate in NASH livers (Ma et al., 2016). We treated hepatocytes with PA and LA for 24 h. One hour prior to the FFA challenge, hepatocytes were treated with 0.2 and 0.4 NTP NU/mL. Treatments with PA and LA markedly increased hepatocyte lipid contents, as demonstrated by Oil red O staining (Figures 1A,B). These increases were significantly inhibited by treatment with NTP (Figures 1A,B). Cellular lipids were also examined by measuring the cellular triglyceride (TG) content. Consistently, PA and LA treatments increased the TG content in primary hepatocytes, whereas NTP treatment significantly decreased PA- and LA-induced increases in TG content (Figure 1C). These findings demonstrate that NTP inhibits lipid accumulation in hepatocytes. In addition, NTP also showed a cytoprotective effect on PA- and LA-treated hepatocytes. The decreased cell viabilities by PA and LA treatments were recovered by NTP treatment (Supplementary Figure S2).

### NTP Treatment Induces Changes in the Expression of Genes Associated With Mitochondrial Function in Hepatocytes

**FIGURE 2:**
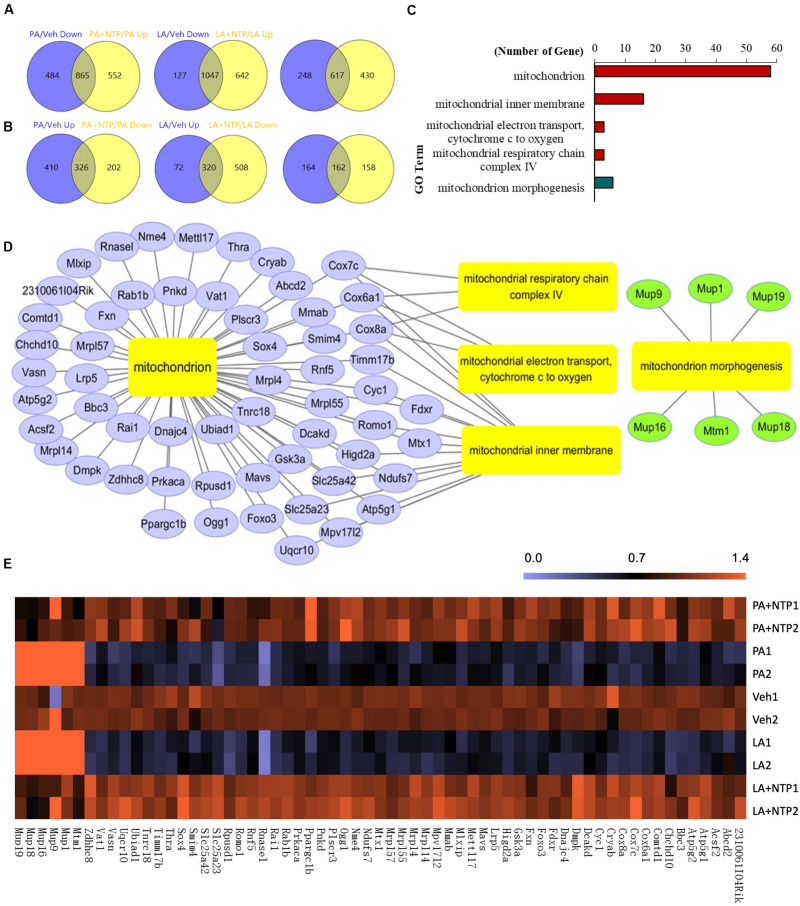
RNA-Seq analysis of NTP-regulated genes in PA- and LA-treated hepatocytes. Primary hepatocytes were pretreated with 0.4 NU/ml NTP for 1 h, followed by treatment with 200 μM palmitate (PA) or 12 μM linoleate (LA) for an additional 24 h. RNA-Seq was applied to analyze the differentially expressed genes (DEGs) regulated by NTP. **(A)** Venn diagrams showing the numbers of genes upregulated by NTP in PA- **(left)** and LA-treated **(middle)** cells, and the overlap **(right)** of both PA- and LA-treated cells. **(B)** Venn diagrams showing the numbers of genes downregulated by NTP in PA- **(left)** and LA-treated **(middle)** cells, and the overlap **(right)** of both PA- and LA-treated cells. **(C)** Gene Ontology (GO) analysis shows mitochondrion-related GO terms regulated by NTP. The overlap genes from **(A) (right)** and **(B) (right)** were used for GO analysis. Red bars represent GO terms associated with upregulated genes, and green bar represents GO terms associated with downregulated genes. **(D)** Network analysis for the mitochondrion-related genes regulated by NTP in PA- and LA-treated cells. **(E)** Heat map for mitochondrion-related genes regulated by NTP.

Next, we comprehensively analyzed the changes in gene expression that may underlie the protective effect of NTP on lipid accumulation in hepatocytes. RNA sequencing (RNA-Seq) identified 1,349 genes that were downregulated by PA treatment, of which the expression of 865 was recovered by NTP treatment (Figure 2A, left). Similarly, of the 1,174 genes downregulated by LA treatment, the expression levels of 1,047 of these were recovered by NTP treatment (Figure 2A, middle). A total of 617 genes were downregulated by both PA and LA treatments and restored by NTP (Figure 2A, right). We also identified 736 genes that were upregulated by PA treatment, of which the expression of 326 was decreased by NTP treatment (Figure 2B, left); 392 genes were upregulated by LA treatment, of which the expression of 320 was decreased by NTP treatment (Figure 2B, middle). In addition, 162 genes were upregulated by PA and LA treatments and reversed by NTP (Figure 2B, right). To determine which signaling pathways were affected by NTP, we performed a Gene Ontology (GO) analysis using the genes changed by NTP in PA- and LA-treated hepatocytes. The GO analysis revealed that a large number of these genes were related to mitochondria, as well as the mitochondrial inner membrane, mitochondrial electron transport, cytochrome *c* to oxygen, mitochondrial respiratory chain complex IV, and mitochondrial morphogenesis (Figure 2C). This suggests that NTP may affect the mitochondrial functions altered by FFAs by modifying the expression of these genes, including that coding for PGC-1β, which is associated with mitochondrial biogenesis and lipolysis, and for oxoguanine glycosylase 1 (OGG1), which is involved in the repair of mitochondrial DNA damage (Figures 2D,E).

### NTP Prevents Mitochondrial Damage and Enhances Mitochondrial Function in Hepatocytes

**FIGURE 3:**
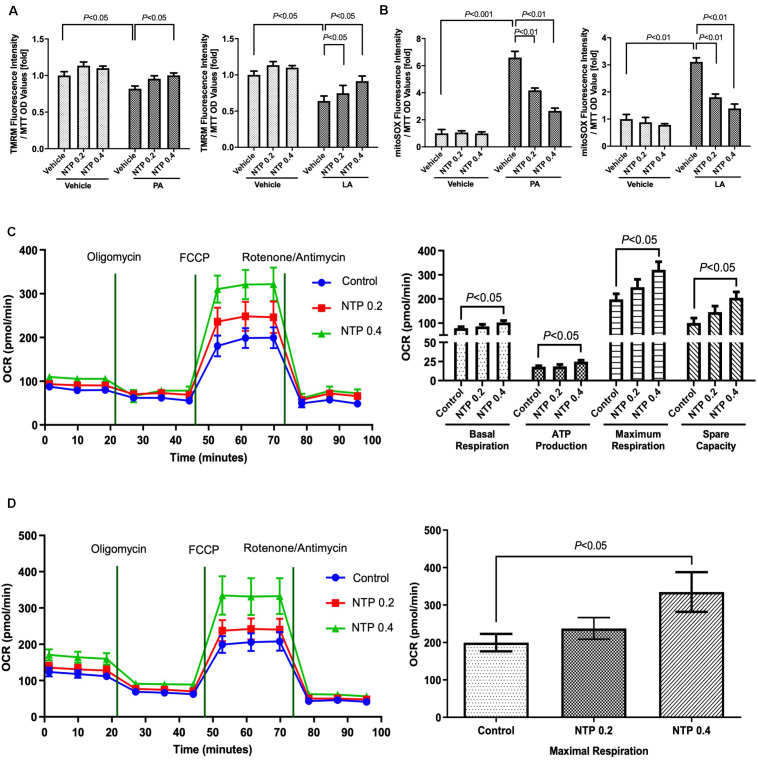
Effects of NTP on mitochondrial function in primary hepatocytes. Primary hepatocytes were pretreated with NTP (0.2 or 0.4 NU/mL) for 1 h, followed by treatment with 200 μM palmitate (PA) or 12 μM linoleate (LA) for an additional 24 h. **(A)** Mitochondrial membrane potential was measured by tetramethylrhodamine (TMRM) ethyl ester perchlorate fluorescence. The values are normalized to MTT OD values, and expressed as fold change compared with vehicle controls. **(B)** Mitochondrial ROS analysis was examined by MitoSox red. The values are normalized to MTT OD values, and expressed as fold change compared with vehicle controls. **(C)** Mitochondrial respiration was measured by an XF24 extracellular flux analyzer. Primary hepatocytes were treated with NTP (0.2 or 0.4 NU/ml) for 24 h before measuring oxygen consumption rates (OCRs). **(Left)** Kinetics of OCRs after sequential compound injections; **(right)** bar charts highlighting the differences in basal respiration, ATP production, maximal respiration, and spare respiratory capacity. The OCR values were normalized to total protein concentrations. **(D)** Fatty acid oxidation (FAO) was measured by the Agilent Seahorse XF PA-BSA FAO assay. Primary hepatocytes were treated with NTP (0.2 or 0.4 NU/ml) for 8 h, and then the medium was changed to substrate-limited medium containing NTP overnight, followed by OCR measurement. (Left) Kinetics of OCR; (right) bar charts highlighting the differences in maximal respiration. The OCR values were normalized to total protein concentrations. Data are represented as mean ± SEM of triplicate. Similar results were obtained in three independent experiments. Representative results are shown.

To investigate how NTP protects against mitochondrial dysfunction caused by FFAs, we measured MMPs and mtROS production in primary hepatocytes. PA and LA treatment markedly reduced MMPs, which was prevented by NTP treatment (Figure 3A). Moreover, PA and LA treatment increased mtROS production, which was significantly suppressed by NTP treatment (Figure 3B). Next, we used a Seahorse bioanalyzer to examine the effect of NTP on the mitochondrial electron transport chain reaction and oxidative phosphorylation. The higher concentration of NTP (0.4 NU/mL) significantly increased the oxygen consumption rate (OCR) during basal respiration, ATP production, and maximal respiration, as well as the spare respiratory capacity (Figure 3C). We also found that NTP treatment similarly increased β-oxidation in hepatocytes (Figure 3D). These findings suggest that NTP reduces mitochondrial damage and suppresses mtROS production while promoting mitochondrial electron transport chain reactions, oxidative phosphorylation, and β-oxidation.

### NTP Treatment Promotes Mitochondrial Turnover in Hepatocytes

**FIGURE 4:**
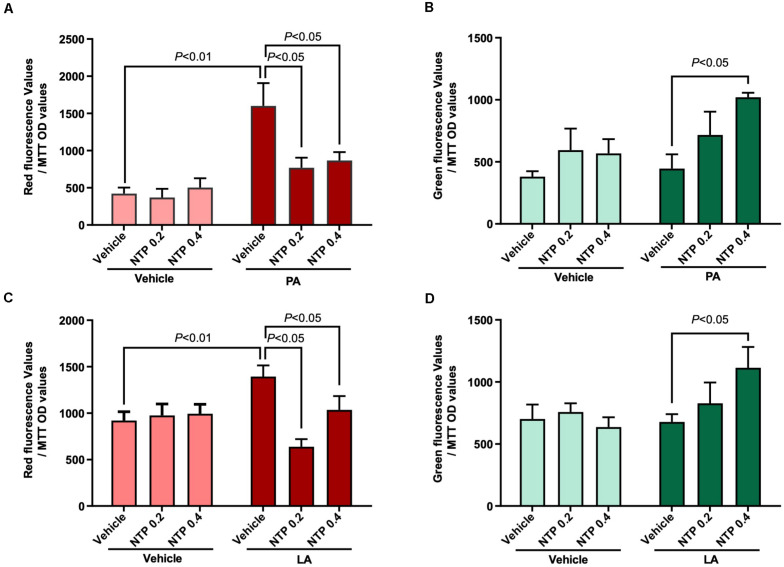
Effects of NTP on mitochondrial turnover in HepG2 cells. Mitochondrial turnover was analyzed by measuring green and red fluorescence intensities in HepG2 cells stably transfected with pMitoTimer (MitoTimer-HepG2 cells). MitoTimer-HepG2 cells were pretreated with NTP (0.2 or 0.4 NU/ml) for 1 h, followed by treatment with 200 μM palmitate (PA) or 12 μM linoleate (LA) for an additional 24 h. Green (476 nm) and red (589 nm) fluorescence intensities were measured by the microplate reader. The values were normalized to MTT OD values. **(A)** Red and **(B)** green fluorescence intensities in PA-treated cells. **(C)** Red and **(D)** green fluorescence intensities in LA-treated cells. Data are represented as mean ± SEM (*n* = 6). Similar results were obtained in two independent experiments. Representative results are shown.

Mitochondrial quality control is essential for maintaining optimal mitochondrial function and is regulated by the balance between mitochondrial biogenesis and destruction, which can be predicted by evaluating mitochondrial turnover (Hernandez et al., 2013; Gottlieb and Stotland, 2015). We generated HepG2 cells stably expressing the Timer protein in mitochondria (MitoTimer-HepG2 cells). In these cells, newly synthesized Timer exhibits green fluorescence that turns to red after 48 h, such that measurements of red and green fluorescence can be used to estimate mitochondrial turnover. PA and LA treatment enhanced the intensity of the red fluorescence signal, indicating an increase in the number/amount of aged mitochondria (Figures 4A,C). Notably, NTP treatment significantly inhibited the increase in red fluorescence and increased the intensity of green fluorescence (Figures 4B,D), suggesting that NTP treatment suppresses PA- and LA-induced mitochondrial aging and promotes mitochondrial biogenesis.

### NTP Treatment Induces AMPK Activation and PGC-1β Expression in Hepatocytes

**FIGURE 5:**
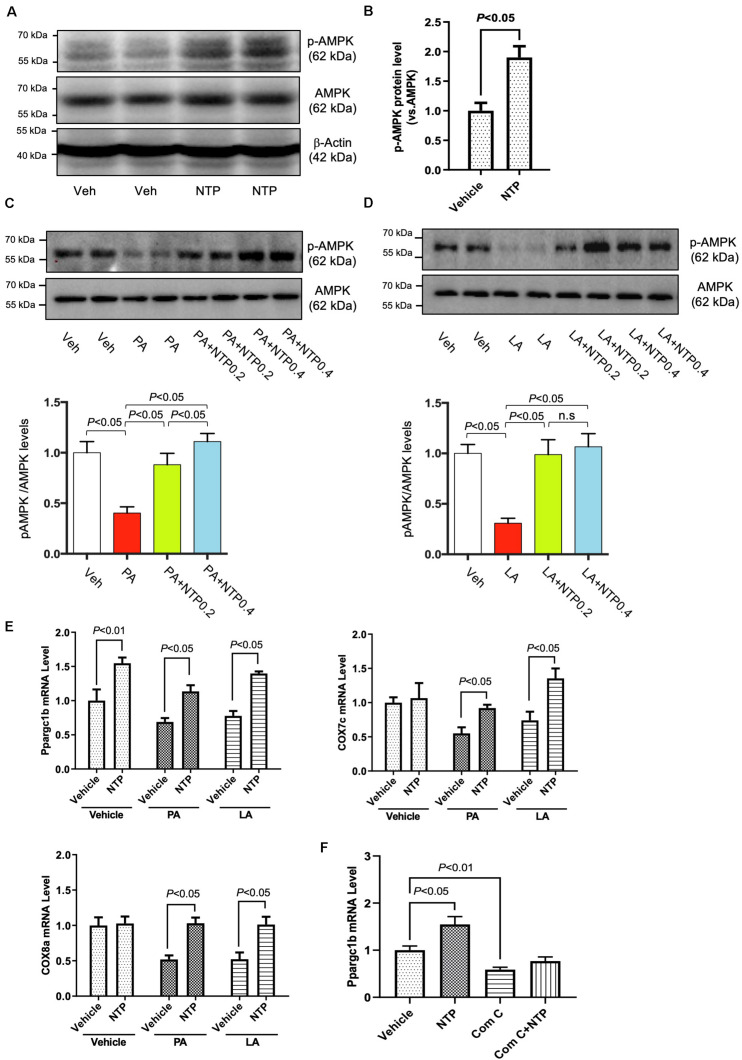
Effects of NTP on AMPK phosphorylation and *Ppargc1b* mRNA expression. **(A)** Primary hepatocytes were treated with NTP (0.2 NU/ml) for 3 h. Phosphorylated AMPK (p-AMPK) and total AMPK protein expression were examined by Western blotting. **(B)** Densitometric quantification of AMPK phosphorylation, normalized to total AMPK. Results were obtained from 3 independent experiments. **(C,D)** Primary hepatocytes were pretreated with NTP (0.2 or 0.4 NU/mL) for 1 h, followed by treatment with **(C)** 200 μM palmitate (PA) or **(D)** 12 μM linoleate (LA) for an additional 24 h. p-AMPK and total AMPK protein expression were examined by Western blotting. Representative western blottings were shown. Densitometric quantification of AMPK phosphorylation, normalized to total AMPK, and expressed as fold change compared with vehicle controls. Four biologically independent samples/group were used for the quantification. **(E)** Primary hepatocytes were pretreated with NTP (0.4 NU/mL) for 1 h, followed by treatment with 200 μM PA or 12 μM LA for an additional 24 h. *Ppargc1b*, *Cox7c*, and *Cox8a* mRNA levels were examined by quantitative real-time PCR. **(F)** Primary hepatocytes were treated with vehicle (0.05%DMSO) or compound C (5 μM), with or without NTP for 24 h, and *Ppargc1b* mRNA levels were examined by quantitative real-time PCR. Data are represented as mean ± SEM of triplicate. Similar results were obtained in two independent experiments. Representative results are shown. Veh, vehicle; n.s., not significant.

AMPK is a multifunctional intracellular kinase that negatively regulates lipid synthesis and accelerates lipid degradation by enhancing mitochondrial β-oxidation (Herms et al., 2015). AMPK phosphorylation was observed 3 h after hepatocytes were treated with NTP (Figures 5A,B), demonstrating that NTP activates AMPK. Moreover, the levels of AMPK phosphorylation decreased by PA and LA treatment were restored to the normal levels by 0.2 NU/mL of NTP and further increased by 0.4 NU/mL of NTP (Figures 5C,D). AMPK activation is associated with fatty acid oxidation (FAO) via the induction of PPARα and PGC-1 (Lee et al., 2006). PGC-1β coactivates PPARγ and maintains mitochondrial biogenesis (Scarpulla, 2008). Our RNA-Seq results revealed a decrease in the expression of PGC-1β in hepatocytes treated with PA and LA, which was reversed by NTP treatment (Figure 2E). We validated these results with quantitative real-time PCR (qPCR), revealing that NTP treatment alone increased PGC-1β expression and prevented PA- and LA-induced reduction in hepatocytes; NTP treatment also increased *Cox7c* and *Cox8a* expression in PA- and LA-treated hepatocytes (Figure 5E). The NTP-mediated induction of PGC-1β was found to be mediated by AMPK, as the increase was inhibited by treating hepatocytes with an AMPK inhibitor (compound C) (Figure 5F).

### Anti-steatotic Effect of NTP Is Mediated Through AMPK and PGC-1β in Hepatocytes

**FIGURE 6:**
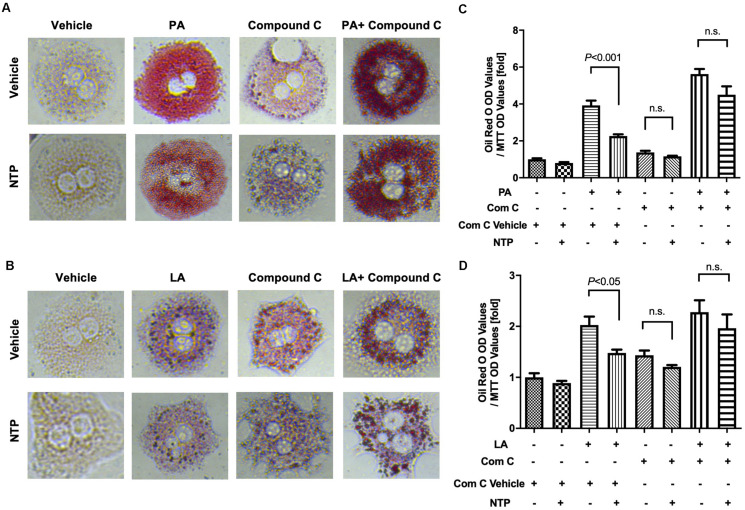
Role of AMPK in NTP-mediated suppression of lipid accumulation in hepatocytes. Primary hepatocytes were pretreated with vehicle (0.05%DMSO) or compound C (Comp C, 5 μM), with or without NTP for 1 h, followed by treatment with 200 μM palmitate (PA) **(A,C)** or 12 μM linoleate (LA) **(B,D)** for an additional 24 h. Cellular lipid accumulation was evaluated by Oil red O staining. **(A,B)** Representative microscopic images of Oil red O staining; **(C,D)** lipid contents were quantified by OD at 500 nm. The values were normalized to MTT OD values, and expressed as fold change compared with vehicle controls. Data are represented as mean ± SEM (*n* = 6). Similar results were obtained in two independent experiments. Representative results are shown. n.s., not significant.

**FIGURE 7:**
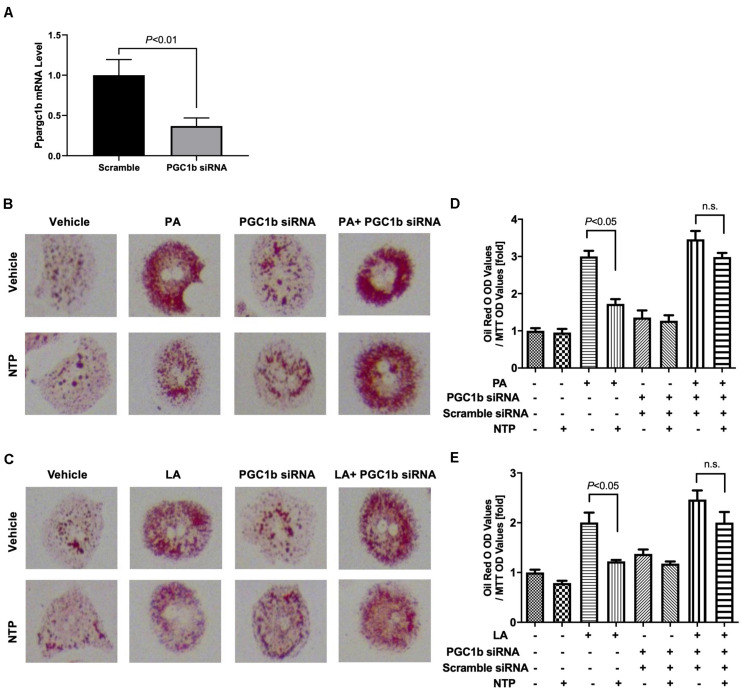
Contribution of PGC-1β to NTP-mediated suppression of lipid accumulation in hepatocytes. Primary hepatocytes were incubated with scramble siRNA or siRNA for *Ppargc1b* for 24 h. **(A)** Expression of *Ppargc1b* mRNA was shown. After silencing *Ppargc1b*, hepatocytes were treated with or without NTP for 1 h. Subsequently, the cells were treated with 200 μM palmitate (PA) **(B,D)** or 12 μM linoleate (LA) **(C,E)** for an additional 24 h. Cellular lipid accumulation was evaluated by Oil red O staining. **(B,C)** Representative microscopic images of Oil red O staining; **(D,E)** lipid contents were quantified by OD at 500 nm. The values were normalized to MTT OD values, and expressed as fold change compared with vehicle controls. Data are represented as mean ± SEM (*n* = 6). Similar results were obtained in two independent experiments. Representative results are shown. n.s., not significant.

To investigate whether NTP-mediated inhibition of lipid droplets is mediated through AMPK, we treated hepatocytes with the AMPK inhibitor and examined whether the anti-steatotic effect of NTP was diminished. Compound C, a cell-permeable AMPK inhibitor, was used to block AMPK activation in this study (Liu et al., 2014). The AMPK inhibitor Compound C alone slightly increased lipid accumulation in hepatocytes, but it did not reach statistically significant levels (Figures 6A–D). Interestingly, the anti-steatotic effect of NTP was not observed in the AMPK inhibitor-treated hepatocytes (Figures 6A–D), suggesting that NTP inhibits FFA-induced lipid accumulation via AMPK. Because AMPK induces the expression of PGC-1β, which regulates lipid homeostasis and mitochondrial biogenesis (Villena, 2015), we examined whether PGC-1β plays a role in the NTP-mediated anti-steatotic effect. We used small interfering RNAs (siRNAs) to silence PGC-1β expression in primary hepatocytes (Figure 7A) and then treated with PA, LA, and/or NTP. Similar to the results with the AMPK inhibitor, NTP failed to suppress the PA- and LA-induced lipid accumulation in hepatocytes with PGC-1β silencing (Figures 7B–E). These results suggest that NTP inhibits lipid accumulation by inducing PGC-1β expression.

### NTP Treatment Maintains Mitochondrial Turnover Through AMPK

**FIGURE 8:**
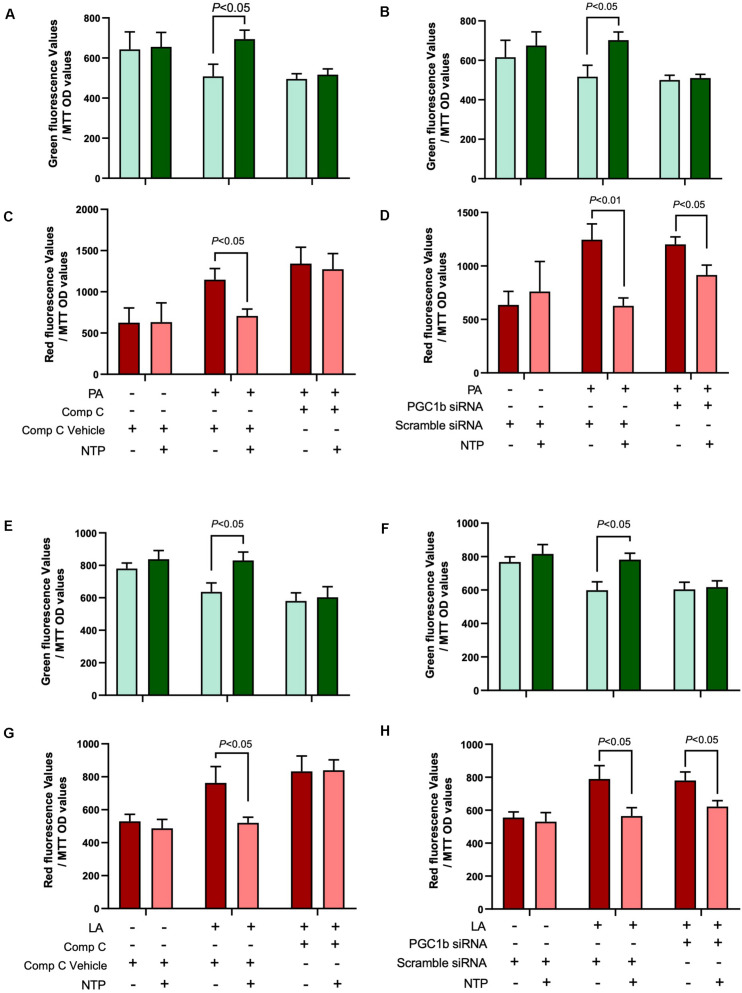
Roles of AMPK and PGC-1β in NTP-mediated mitochondrial turnover. Mitochondrial turnover was analyzed by measuring green and red fluorescence intensities in MitoTimer-HepG2 cells. AMPK activity was inhibited by compound C, and *Ppargc1b* was silenced by the transfection of siRNAs. **(A,B,E,F)** MitoTimer-HepG2 cells were pretreated with vehicle (0.05%DMSO) or compound C (Comp C, 5 μM), or **(C,D,G,H)** were incubated with scramble siRNA or siRNA for *Ppargc1b* for 24 h, and then treated with NTP (0.4 NU/ml) for 1 h, followed by treatment with 200 μM palmitate (PA) or 12 μM linoleate (LA) for an additional 24 h. Green (476 nm) and red (589 nm) fluorescence intensities were measured by the microplate reader. The values were normalized to MTT OD values. **(A,C)** Red and **(B,D)** green fluorescence intensities in PA-treated cells. **(E,G)** Red and **(F,H)** green fluorescence intensities in LA-treated cells. Data are represented as mean ± SEM (*n* = 6). Similar results were obtained in two independent experiments. Representative results are shown.

Because AMPK and PGC-1β regulate mitochondrial biogenesis, we next investigated whether AMPK and PGC-1β contribute to mitochondrial turnover maintained by NTP treatment in hepatocytes. We did not observe an increase in green fluorescence and decrease in red fluorescence in NTP- and FFA-treated hepatocytes when cells were treated with the AMPK inhibitor Compound C (Figures 8A,C,E,G), suggesting that the beneficial effect of NTP on aging and mitochondrial synthesis in PA- and LA-treated hepatocytes is mediated by AMPK. By contrast, when PGC-1β was silenced, green fluorescence did not increase, but red fluorescence slightly decreased with NTP treatment. This suggests that the beneficial effect of NTP on mitochondrial aging is only partially mediated by PGC-1β but that PGC-1β contributes to the enhancement of mitochondrial synthesis (Figures 8B,D,F,H). Thus, although NTP-mediated AMPK activation is important for mitochondrial turnover, the induction of PGC-1β is more important for mitochondrial biogenesis than for preventing mitochondrial aging.

### NTP-Induced JNK Inhibition Can Suppress FFA-Induced Lipid Accumulation but Independent of AMPK

**FIGURE 9:**
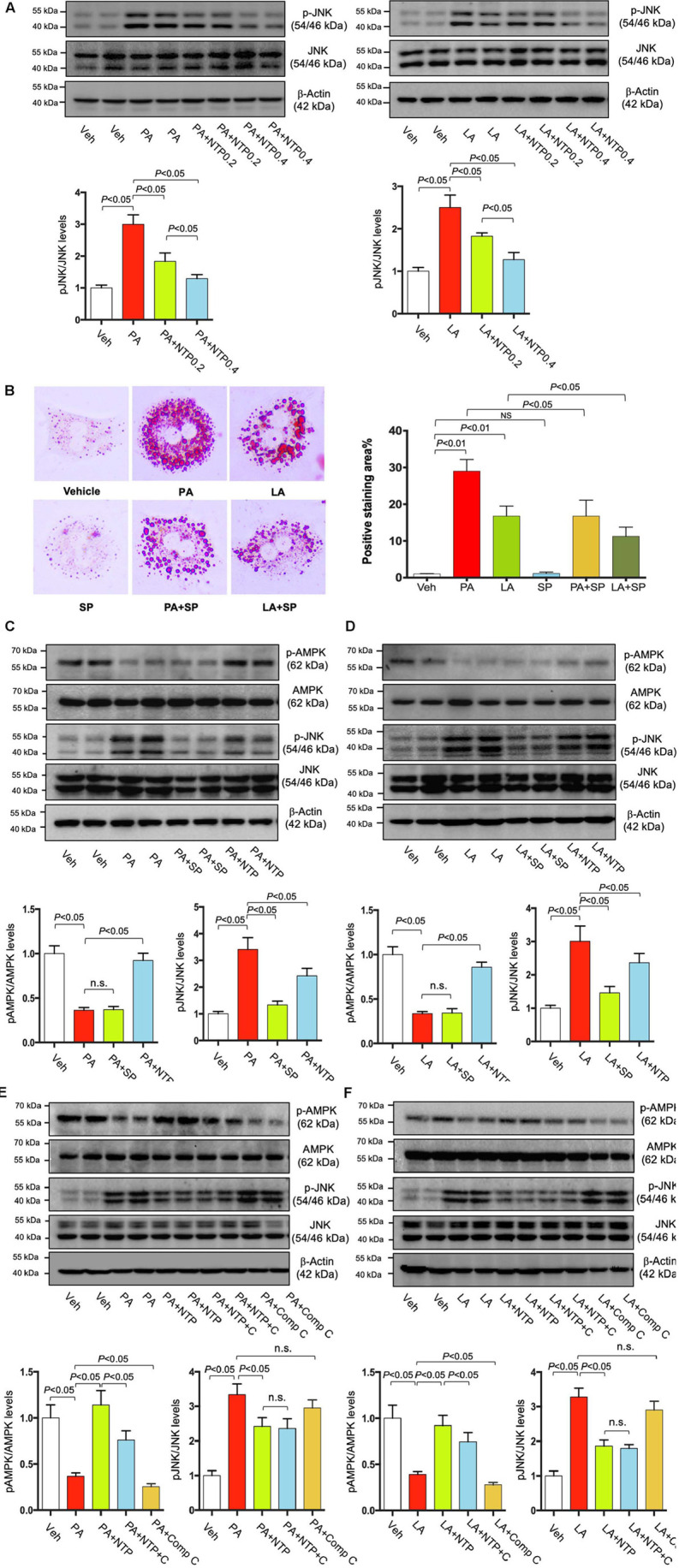
Effects of NTP and JNK inhibition on lipid accumulation in hepatocytes. **(A)** Primary hepatocytes were pretreated with NTP (0.2 or 0.4 NU/mL) for 1 h, followed by treatment with 200 μM palmitate (PA) or 12 μM linoleate (LA) for an additional 24 h. p-JNK and total JNK protein expression were examined by Western blotting. Representative western blottings were shown. Densitometric quantification of JNK phosphorylation, normalized to total JNK, and expressed as fold change compared with vehicle controls. Four biologically independent samples/group were used for the quantification. **(B)** Primary hepatocytes were pretreated with SP600125 (20 μM) for 1 h, followed by treatment with 200 μM PA or 12 μM LA for an additional 24 h. Cellular lipid accumulation was examined by Oil red O staining. Representative microscopic images and quantification. Data are presented as the means ± standard errors of the means of 10 high power fields (×200). **(C–F)** Primary hepatocytes were pretreated with NTP (0.4 NU/mL), SP600125 (SP, 20 μM), or Compound C (C, Comp C, 5 μM) for 1 h, followed by treatment with 200 μM PA or 12 μM LA for an additional 24 h. p-AMPK, total AMPK, p-JNK and total JNK protein expression were examined by Western blotting. Representative western blottings were shown. Densitometric quantification of phosphorylation of AMPK and JNK, normalized to total AMPK and JNK, respectively, and expressed as fold change compared with vehicle controls. Four biologically independent samples/group were used for the quantification. Veh, vehicle; n.s., not significant.

Given that our previous study showed that NTP inhibited IL-1β-induced c-Jun N-terminal kinase (JNK) activation (Zhang et al., 2014), and PA is known to activate JNK (Seki et al., 2012), we examined whether JNK inhibition is associated with the NTP-mediated effect on FFA-induced lipid accumulation. FFA (both PA and LA) induced JNK phosphorylation, and NTP inhibited PA/LA-induced JNK phosphorylation (Figure 9A). We found that SP600125, a JNK inhibitor, inhibited PA/LA-induced lipid accumulation (Figure 9B). These findings suggest that NTP inhibition of JNK could be associated with NTP-mediated reduction of PA/LA-induced lipid accumulation in hepatocytes.

Next, we investigated whether NTP-induced AMPK phosphorylation is mediated through JNK inhibition. First, we inhibited JNK using SP600125, but we did not find the effect of JNK inhibition in AMPK phosphorylation (Figures 9C,D). We then inhibited AMPK phosphorylation using Compound C, but we also did not find the effect of AMPK inhibition on JNK phosphorylation (Figures 9E,F). These data suggest that NTP has the ability to promote AMPK phosphorylation as well as inhibit JNK phosphorylation, both of which can suppress FFA-induced lipid accumulation. However, the effects of AMPK and JNK could be independent and do not affect each other.

## Discussion

Non-alcoholic fatty liver disease is a hepatic manifestation of metabolic syndrome, which is associated with obesity, type 2 diabetes, and dyslipidemia as well as cardiovascular disease (Saltiel and Olefsky, 2017). The accumulation of lipids in hepatocytes induces ROS production, mitochondrial damage, and apoptosis signaling (Koyama and Brenner, 2017). Mitochondria are the major organelles for energy production, oxidative respiration, glucose metabolism, and lipid homeostasis, including β-oxidation. As NAFLD progresses, mtROS production and β-oxidation inhibition are increased, which promotes further mitochondrial damage and dysfunction in a positive feedback loop (Sunny et al., 2017), thereby enhancing hepatic steatosis, hepatocyte death, and inflammation (Koliaki et al., 2015). Thus, mitochondrial damage and dysfunction likely underlie NAFLD progression.

In the present study, we induced mitochondrial damage in hepatocytes with PA and LA, the most abundant hepatotoxic FFAs in NAFLD livers (Ma et al., 2016), which led to accumulations of lipid droplets in the cytoplasm, decreased MMP, and increased mtROS production. Notably, these effects were reversed by NTP. Our data corroborate the findings from a previous study showing that NTP was similarly protective against amyloid protein β-induced neuronal cell damage (Fang et al., 2017). Together, these studies provide evidence that NTP protects against mitochondrial dysfunction and maintains mitochondrial quality control.

Our comprehensive gene analysis demonstrated that the expression of a number of genes in hepatocytes was altered by PA and LA treatment and that these alterations were mitigated by NTP treatment. Our bioinformatics analysis revealed that NTP affected the expression of a variety of genes associated with mitochondrial function and morphogenesis. For example, PA and LA treatments reduced the expression of PGC-1β, which was restored by NTP treatment. In skeletal muscle, PGC-1β and PGC-1α play roles in mitochondrial biogenesis to increase mitochondrial mass and oxidative respiration: PGC-1β helps to regulate the basal condition of mitochondria and lipid homeostasis in response to a high-calorie diet, whereas PGC-1α is induced by exercise or caloric restriction to regulate gluconeogenic gene expression (Villena, 2015). In the liver, both PGC-1α and PGC-1β regulate mitochondrial biogenesis through the expression of mitochondrial genes. Intriguingly, liver-specific PGC-1β-deficient mice exhibit increased hepatic TG content when fed a high-fat/high-carbohydrate diet (Lelliott et al., 2006; Vianna et al., 2006; Sonoda et al., 2007; Chambers et al., 2012). In these mice, impaired mitochondrial function and β-oxidation, corresponding to reduced expression of genes involved in β-oxidation and oxidative phosphorylation pathways, are associated with hepatic steatosis. The reduced expression of PGC-1β in hepatocytes treated with LA and PA in the present study is consistent with its role in mitochondrial biogenesis and lipid homeostasis. Moreover, NTP prevented this decrease in expression and PA- and LA-induced mitochondrial aging (or reduced elimination of aged mitochondria) and accelerated mitochondrial biogenesis, as demonstrated by increased green fluorescence in MitoTimer-HepG2 cells.

In the present study, we identified a potential mechanism involving AMPK in NTP-mediated inhibition of FFA-induced lipid accumulation. AMPK plays a role in cellular energy homeostasis via activating glucose and fatty acid uptake and oxidation and for which numerous studies have documented roles in regulating mitochondrial function and biogenesis (Reznick and Shulman, 2006; Marin et al., 2017; Herzig and Shaw, 2018). Thus, increasing the activity of AMPK has been viewed as a viable therapeutic strategy to improve NAFLD (Smith et al., 2016). We found that NTP promotes AMPK phosphorylation, suggesting that the effects of NTP on suppressing lipid accumulation and maintaining mitochondrial function in hepatocytes are mediated through AMPK activation. AMPK has also been reported to induce autophagy either directly or indirectly via the suppression of mTORC1 (Mihaylova and Shaw, 2011), which may reduce hepatic intracellular lipid droplet deposition in hepatocytes by promoting fat droplet degradation (Liu and Czaja, 2013). Therefore, the reduction of lipid droplet deposition by NTP may involve autophagy mediated by AMPK. Further investigations are needed to clarify this mechanism. The activation of JNK and translocation into mitochondria is crucial for lipotoxicity-induced ROS production and apoptosis (Win et al., 2015; Hirsova et al., 2016). We previously showed that NTP inhibited TNFα and IL-1β-induced JNK activation and hepatocyte apoptosis (Zhang et al., 2014). JNK inhibition is another potential mechanism by which NTP prevents mitochondrial damage and lipid accumulation. We attempted to identify the crosstalk between AMPK and JNK. However, we could not find the association between AMPK activation and JNK inhibition. We suggest that NTP inhibits lipid accumulation through AMPK activation and JNK inhibition, but these pathways independently regulate lipid accumulation.

This study has several limitations. NTP is a well-established drug that is a non-protein extract of vaccinia virus-infected inflamed rabbit skin and has been used to treat chronic pain in Japan and China for several decades. Because NTP is not a single active ingredient drug and contains various molecules, including nucleic acids, amino acids, and sugars (unpublished observations), the crucial biological active components have not been determined. In this study, we have identified AMPK, PGC-1β, mitochondria, and JNK are the targets of NTP. Moreover, previous studies have also determined NTP can inhibit NF-κB, p38, ERK1/2, HIF-1α, and promote PI3K and AKT activation and brain-derived neurotrophic factor induction as the targets of NTP (Zhang et al., 2014; Nishimoto et al., 2016; Fang et al., 2017, 2019; Matsuoka et al., 2018; Sakai et al., 2018; Zheng et al., 2018). We and others have not determined the precise molecular mechanisms of how NTP affects those factors. NTP contains multiple bioactive components, which include adenosine and γ-aminobutyric acid (GABA) (Yao et al., 2020). Increased extracellular adenosine concentration has been reported to induce AMPK phosphorylation (Aymerich et al., 2006). Another study demonstrated that GABA receptor can interact with AMPK, suggesting the effect of GABA in AMPK activity (Jiang et al., 2018). In line with these observations, we speculate that adenosine and GABA in NTP may be involved in AMPK activation. Lastly, our data were generated by *in vitro* culture experiments, and additional studies are needed to determine whether NTP has a beneficial effect to suppress fatty liver disease in animal models *in vivo*.

In summary, the results from the present study demonstrate that NTP has anti-steatotic effects in hepatocytes, which involve the activation of AMPK, induction of PGC-1β, mitochondrial biogenesis, and maintenance of mitochondrial function. These findings support the use of NTP as a novel approach for the treatment of NAFLD. Notably, individuals with chronic pain that can be treated with NTP often overlap with the population of patients with metabolic syndrome (Toda et al., 2000; Melissas et al., 2003; Isonaka et al., 2013; Masuguchi et al., 2014). Whereas many analgesic agents, such as non-steroidal anti-inflammatory drugs, are generally hepatotoxic, this and a previous study (Zhang et al., 2014) demonstrate that NTP is hepatoprotective. *In vivo* preclinical studies are needed to validate the effectiveness of NTP on NAFLD. Nevertheless, our study proposes a novel molecular mechanism for the protective effect of NTP in hepatosteatosis and provides a basis for the potential repurposing of NTP for the treatment of NAFLD.

## Data Availability Statement

The datasets generated for this study can be found in the RNA-seq; data have been uploaded to the GEO (accession no. GSE132251).

## Ethics Statement

The animal study was reviewed and approved by the IACUC of Cedars-Sinai Medical Center.

## Author Contributions

QW, MN, and ES designed the experiments and interpreted the data. QW, ZW, MX, WT, I-FH, AS, and JK performed the experiments and analyzed the data. QW performed the statistical and bioinformatics analysis. QW and ES performed the statistical analysis and wrote the manuscript. PL, MN, RG, and ES commented on the study and revised the manuscript. ES obtained the funding. All authors contributed to the article and approved the submitted version.

## Conflict of Interest

MN is an employee of Nippon Zoki Pharmaceutical Co., Ltd. The study was supported by a research grant from Nippon Zoki Pharmaceutical Co., Ltd. The funder had no role in the study design, data collection and analysis, decision to publish, or preparation of the manuscript. The remaining authors declare that the research was conducted in the absence of any commercial or financial relationships that could be construed as a potential conflict of interest. The handling editor declared a past co-authorship with several of the authors ZW, WT, and ES.
