# Supplementary material for: Neurotropin Inhibits Lipid Accumulation by Maintaining Mitochondrial Function in Hepatocytes via AMPK Activation
Source: Front Physiol. 2020 Aug 6;11:950. doi: 10.3389/fphys.2020.00950 (PMC7424056; doi:10.3389/fphys.2020.00950)
Supplement: Supplementary file 1 [file Data_Sheet_1.pdf]

Supplementary Material

1.1 Supplementary Figures

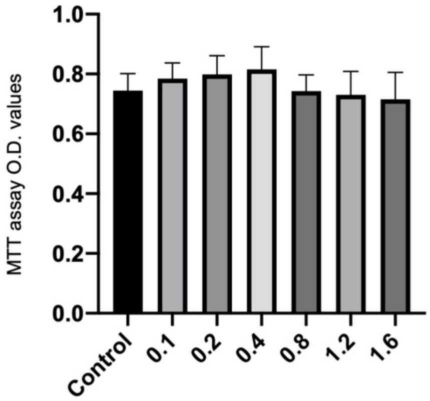

Supplementary Figure 1. Evaluation of toxicity of NTP on primary mouse hepatocytes. Primary hepatocytes were treated with Vehicle or NTP (0.1, 0.2, 0.4, 0.8, 1.2, or 1.6 NU/mL) for 24 h. Hepatocyte viability was examined by MTT assays.

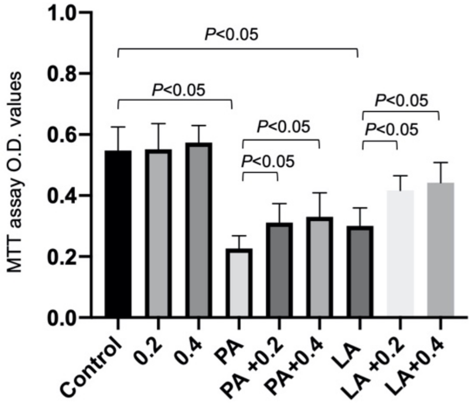

Supplementary Figure 2. Effects of NTP on cell viability in PA- and LA-treated hepatocytes.

Primary hepatocytes were pretreated with NTP (0.2 or 0.4 NU/mL) for 1 h, followed by treatment with 200 μM palmitate (PA) or 12 μM linoleate (LA) for an additional 24 h. Hepatocyte viability was examined by MTT assays.
